# Linking Safety-Specific Leader Reward and Punishment Omission to Safety Compliance Behavior: The Role of Distributive Justice and Role Ambiguity

**DOI:** 10.3389/fpubh.2022.841345

**Published:** 2022-03-16

**Authors:** Lin Liu, Qiang Mei, Anders Skogstad, Jinnan Wu, Suxia Liu, Meng Wang

**Affiliations:** ^1^School of Management, Jiangsu University, Zhenjiang, China; ^2^School of Management Science and Engineering, Anhui University of Technology, Ma'anshan, China; ^3^Department of Psychosocial Science, University of Bergen, Bergen, Norway; ^4^School of Business, Anhui University of Technology, Ma'anshan, China

**Keywords:** safety-specific leader reward omission, safety-specific leader punishment omission, distributive justice, role ambiguity, safety compliance, laissez-faire leadership

## Abstract

**Background:**

Although positive safety leadership has attracted increasingly academic and practical attention due to its critical effects on followers' safety compliance behavior, far fewer steps have been taken to study the safety impact of laissez-faire leadership.

**Objective:**

This study examines the relationships between safety-specific leader reward and punishment omission (laissez-faire leadership) and followers' safety compliance, and the mediations of safety-specific distributive justice and role ambiguity.

**Methods:**

On a two-wave online survey of 307 workers from high-risk enterprises in China, these relationships were tested by structural equations modeling and bootstrapping procedures.

**Results:**

Findings show that safety-specific leader reward omission was negatively associated with followers' safety compliance through the mediating effects of safety-specific distributive justice and role ambiguity. Safety-specific leader punishment omission was also negatively associated with followers' safety compliance through the mediating effect of safety-specific role ambiguity, while safety-specific distributive justice was an insignificant mediator.

**Originality:**

The study addresses and closes more gaps by explaining how two contextualized laissez-faire leadership measures relate to followers' safety behaviors, following the contextualization and matching principles between predictors, mediators and criteria, and by revealing two mechanisms behind the detrimental effects of laissez-faire leadership on safety outcomes.

## Introduction

Despite significant efforts over the past several decades, workplace injuries and accidents have shown to be frequent among national and global firms in high-risk industries, which indicates that occupational safety is still a highly relevant and serious issue worthy of academic attention (1, 2). Among the strongest antecedents of injuries and accidents, increased individual compliance with organizational safety procedures and standards has shown to decrease the probability and number of accidents, injuries, and near-misses (1, 3, 4). Accordingly, two meta-analytic reviews have shown the robust prediction of safety compliance on various safety outcomes (5, 6). Hence, to minimize the direct and consequential losses of accidents, the measures for motivating safety compliance have become focal points for scholars in this field (5–7).

Recently, safety-specific leadership has attracted increasingly academic and practical attention due to its critical effects on followers' safety compliance behavior (8–11) (Appendix A summaries some of safety leadership studies). Since Barling et al. (8) and Zohar (12), extensive studies have supported the positive relationship between safety-specific *transformational leadership* and followers' safety compliance through the mediation of safety climate, safety consciousness and safety attitude (8, 10, 13–16). Similar to transformational leadership, *active transactional leadership* (i.e., leader contingent reward and punishment) has been further substantiated to be an effective way to improve employees' occupational safety compliance (9, 15, 17).

However, compared to transformational and transactional leadership, far fewer steps have been taken to study the safety impact of *laissez-faire leadership*, proposed as a destructive type of leadership (18, 19). Given that most followers are more likely to experience laissez-faire leadership (20), it is relevant and important to examine the potential effect of laissez-faire leadership on followers' safety compliance. Although the negative effects of laissez-faire leadership have been demonstrated in few safety studies conducted in the USA and European countries (15, 17, 21), we believe that further studies, and not the least in China with a different national culture, are important and necessary for testing the generalizability of the link between laissez-faire leadership and followers' safety compliance. Moreover, we notice that previous studies have used a relative generalized and uncontextualized measure of laissez-faire leadership (22), which may prevent us from getting a nuanced understanding of the predictive power of safety-specific laissez-faire leadership behavior on corresponding follower safety-specific behavior in the form of safety compliance (23, 24). Further, and in line with the fact that laissez-faire leadership has been a stepchild in leadership research (25), very few studies have focused on the underlying mechanisms that may explain the detrimental effects of laissez-faire leadership on various attitudinal and behavioral outcomes.

Hence, the current study strives to narrow these gaps by examining the impact of safety-specific laissez-faire leadership on follower safety compliance behavior in Chinese high-risk firms. Based on the fact that no previous study explicitly have measured supervisor's lack of responsiveness to good or poor follower performance, Hinkin and Schriesheim (25) developed two measures of “reward omission” (leader non-reinforcement of follower's good performance) and “punishment omission” (leader non-reinforcement of follower's poor performance), respectively, to capture leader's lack of performance-contingent reinforcements. As predicted, they found that leader reward and punishment omissions were negatively associated with supervisor-rated follower performance. Following these findings, and other well-documented findings on the negative and detrimental impact of laissez-faire leadership (12, 15, 17–19, 21), the present study introduces safety-specific reward and punishment omissions to the occupational safety context, and examines the effects of these two domain-specific laissez-faire leadership styles on followers' safety compliance.

A second purpose of this study is to reveal the mechanisms through which safety-specific laissez-faire leadership influences followers' safety compliance behavior by examining two parallel mediations, i.e., safety-specific distributive justice and safety-specific role ambiguity, respectively. We propose these two mechanisms based on the study by Podsakoff et al. (26) which showed that two main mechanisms explained how leader reward and punishment behaviors influenced follower cognitive and behavioral responses, namely followers' perceptions of fairness (27, 28) and role ambiguity (29, 30), respectively. Hence, we propose that contextualized distributive justice as well as role ambiguity will mediate the effects of superiors' safety-specific reward and punishment omission on followers' safety compliance.

Hence, we aim to contribute significantly to the laissez-faire leadership research field and to the workplace safety literature in several ways. First, as a response to recent calls for more attention to laissez-faire leadership in occupational safety settings (15, 31, 32), we contextualize leader reward and punishment omission measures to, in a nuanced way, better predict the effects of laissez-faire leadership on safety outcomes (33, 34). Accordingly, we contribute to safety as well as laissez-faire leadership research by, for the first time, testing two contextualized leadership omission measures within a safety framework, and matching leader and follower behaviors on congruent levels. Second, the present study is, to our knowledge, the first study to link safety-specific leader reward and punishment omission to follower safety compliance, which contributes substantially to a better understanding of how concrete leadership behaviors are related to followers' concrete safety behaviors. Finally, this study expands upon the literature on workplace safety and laissez-faire by revealing underlying mechanisms, in the present study operationalized as followers' safety-specific justice and safety-specific role ambiguity (35). This study is, to our knowledge, the first study to test the parallel effects of these two mediators in the passive-avoidant leadership—safety behavior relationship, thus contributing substantially to workplace safety research with direct consequences for workplace safety interventions.

## Theory Development

### Reward and Punishment Omission in a Leadership and Safety Context

Given that the omission of leadership may be just as important as its commission, Hinkin and Schriesheim (25) introduced the concepts of leader reward omission and punishment omission which represent two types of interrelated concretized laissez-faire leadership behaviors. Reward omission refers to leader non-reinforcement of what a follower perceives to be his or her good performance. Accordingly, punishment omission refers to leaders' non-reinforcement of what a follower perceives to be his or her poor performance. Given the fact that people may think, perceive and act differently in different contexts (36), several studies have suggested that specifying the context through a frame-of-reference changes responses to scale items and typically improves the prediction of performance outcomes (33, 34, 37), especially when the outcome scale items are on the same level of specificity [cf., the matching principle; see e.g., (38)]. Unlike prior research focusing on relationship between leadership behaviors and safety outcomes without contextualizing leadership measures (8, 14, 15, 21), the present study, thus, relates these two types of leadership omissions by the contextual operationalization of safety-specific leader reward omission (SLRO) and a corresponding safety-specific leadership punishment omission (SLPO), both scales reflecting leaders' lack of motivational efforts toward stimulating and facilitating followers' safety behaviors (16, 32). Our contextualization is consistent with recent studies (10, 39) which have specified the safety context for transformational leadership, stressors, trust, motivation and citizenship behavior. Hence, we argue that such leader reward and punishment omission measures, tailored for the safety-specific context, will yield both higher content and criterion validity, as well as reliability, than those general measures of leadership in predicting employee safety compliance behaviors (34, 37).

### The Influences of SLRO and SLPO on Perceived Safety-Specific Distributive Justice and Safety-Specific Role Ambiguity

Previous studies have established that how leaders administer rewards and punishments, respectively, influences followers' cognitive processes (27, 40). A recent study has shown that leader reward and punishment behaviors (active-approaching leadership) predict follower attitudes, as well as in-role and extra-role behaviors, via two internal mechanisms, namely perceived justice and role ambiguity (26). Distributive justice refers to the degree to which followers perceive rewards and punishments to be proportionally related to performance inputs (41), while role ambiguity refers to a lack of clarity about which demands and expectations to fulfill (30), also including ambiguity regarding the process and the criteria for evaluating one's safety performance (42). In line with the referred findings by Podsakoff and colleagues above, it is reasonable to expect that SLRO and SLPO (two forms reflecting passive-avoidant leadership) will influence followers' safety compliance via two parallel mechanisms, i.e., perceived safety-specific distributive justice and safety-specific role ambiguity.

Logically, followers strongly believe and expect that the rewards and punishments they receive from their leaders should be fitted to their performances. If their leaders administer rewards and punishments on the basis of such an equity rule, they have shown to be perceived as fairer than those administered by leaders who do not allocate rewards/punishments according to followers' good/poor performances (26). Hence, when followers devote their time and energy to comply with organizational safety-related operations, procedures and rules but do not receive corresponding praise, commendations, social approval or monetary rewards, and link this to their perceived safety performance levels (26), they are more likely to perceive a low level of distributive justice or no such justice at all. Similarly, when followers perceive that other employees have been punished because of their poor safety performance, they are prone to perceive this punishment as more distributively just (26, 27). Thus, it is reasonable to expect that leaders who do not respond to followers who violate safety-related operating procedures and rules will be perceived to be unfairer than those leaders responding according to distributive justice. Hence, we hypothesize:

**Hypotheses 1 and 2:** Safety-specific leader reward omission (H1) and punishment omission (H2) are negatively associated with followers' perceived distributive justice.

A second mechanism through which the two context-specific leadership styles may influences follower safety compliance is that of safety-specific role ambiguity. It is by now well-established that leaders who in a systematic way provide positive and negative feedback help to clarify followers' roles in the organization (26, 29). Specifically, leaders' contingent rewards are decisive in this regard, whereas contingent punishments can serve as a strong signal to judge that the performance levels of followers do not meet the leader's expectations (29). In contrast, laissez-faire leadership has found to be a root source of followers' role ambiguity (18, 43). Accordingly, follower perception of leader reward and punishment omissions have been found to reduce role clarity (alternatively, enhance role ambiguity) (25, 44). Therefore, a reasonable hypothesis is that when followers perceive that superiors do not respond to good or poor follower safety performance, they are prone to become confused about what he or she can do to fulfill desired and expected safety outcomes. That is, supervisors' safety-specific reward and punishment omissions will probably lead to safety-specific role ambiguity regarding followers' fulfillment of duties and specific aspects of task completion. Therefore, we hypothesize that:

**Hypotheses 3 and 4:** Safety-specific leader reward omission (H3) and punishment omission (H4) are positively associated with followers' experienced safety-specific role ambiguity.

### The Influences of Perceived Safety-Specific Distributive Justice and Safety-Specific Role Ambiguity on Followers' Safety Compliance

Distributive justice has been demonstrated to positively relate to job satisfaction (45), organizational commitment (45, 46), employee engagement (47), and perceived insider status (48). To our knowledge, it is unclear whether distributive justice relates to employee's safety compliance behaviors. However, previous studies have found that distributive justice is associated with employee behaviors beneficial to organizations such as presenteeism (49), positive gossip behavior (48), and willingness to cooperate (50). Together, these studies highlight the critical role of distributive justice in generating positive cognitive, affective, and behavioral responses within organizations. Hence, we expect that when leaders reward followers relative to their safety performance, as reflected in safety-specific distributive justice, followers will accordingly comply with organizational safety-related operation procedures and rules, leading to the following hypothesis:

**Hypothesis 5:** Followers' perceived safety-specific distributive justice is positively related to their safety compliance.

Safety-specific role ambiguity refers to cases where available information and resources concerning safety roles are unclear or inadequate (39, 44). Accordingly, role theory suggests that role ambiguity will lead to individuals' dissatisfaction with their roles, hesitation over decisions, anxiety and confusion, and decrease their organizational commitment as well as increase their propensity to leave; and, accordingly, result in ineffective performance (39, 44, 51). If employees are confused about their safety duties, this will probably be followed with vague role expectations and a unsureness about how to operate correctly, which probably will lead to a general lack of confidence in superiors and an inefficacy to enact adequate and sufficient safety behaviors (39, 51). Leung et al. (52) showed that safety-related work stressors (e.g., role ambiguity and role conflict) hindered employees' safety performance and triggered accidents, resulting in injuries. More recently, Wang et al. (39) investigated Chinese frontline workers and their safety supervisors and revealed a negative relationship between safety-specific role ambiguity and proactive safety behaviors, including safety compliance. To conclude, we have substantiated that if employees experience role ambiguity in their work execution, they are expected to violate rather than comply with organizational safety procedures and rules. Hence, we hypothesize that:

**Hypothesis 6:** Followers' experienced safety-specific role ambiguity is negatively associated with their safety compliance.

### The Mediating Role of Perceived Safety-Specific Distributive Justice and Safety-Specific Role Ambiguity in the Leader Behavior—Follower Safety Compliance Relationship

Lack of safety leadership is probably an essential disruptive antecedent of safety performance in organizations. Accordingly, we propose that SLRO and SLPO impede followers' perceived safety-specific distributive justice and enhance their experienced of safety-specific role ambiguity. We, further, propose that this will subsequently lead to followers worsened or lacking safety compliance behaviors. Indeed, studies have shown that laissez-faire leadership influences employee outcomes through the mediation of internal cognitive processes (28, 40). Moreover, prior studies support the idea that laissez-faire, as well as other forms of passive-avoidant leadership, influence safety performance through safety-related cognitive processes (6, 9, 16, 21). Further, our two mediation propositions are in line with a meta-analysis which showed that transactional leadership influences employee behaviors via perceived justice and role ambiguity (26). Hence, we propose the following mediation hypotheses:

**Hypothesis 7:** Perceived safety-specific distributive justice mediates the effect of perceived safety-specific leader reward omission (H7a) on follower safety compliance; and, accordingly, the effect of perceived safety-specific leader punishment omission (H7b).
**Hypothesis 8:** Perceived safety-specific role ambiguity mediates the effect of perceived safety-specific leader reward omission (H8a) on follower safety compliance; and, accordingly, the effect of perceived safety-specific leader punishment omission (H8b).

**Figure 1:**
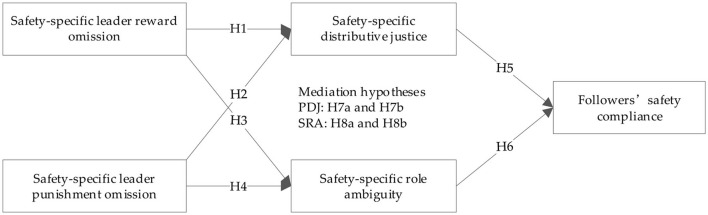
Hypothesized research model.

Figure 1 shows our defined research model and demonstrates that both perceived safety-specific leader reward and punishment omission are expected to affect followers perceived safety-specific distributive justice and safety-specific role ambiguity, and in the following influence their safety compliance, accordingly that perceived safety-specific distributive justice and safety-specific role ambiguity mediate the relationship between leader behaviors and followers' safety compliance behaviors.

## Methods

### Sample and Procedure

This study used a two-wave online questionnaire survey to collect self-reported data for testing the hypotheses. We targeted front-line workers in enterprises located in central China. These enterprises are in four high-risk industries including metal melting, construction, hazardous chemicals and machinery manufacturing. Although we did not recruit the participants from the entire China, they are heterogeneous and representative as regards industry type and firm size. Moreover, our recruitment method (see below) ensured that participants match the research context of workplace safety of this study.

To avoid common method bias, we distributed our online surveys across two different time periods. At the baseline assessment (T1) of the two-wave data collection, participants reported sensitive information, i.e., the frequency with which their superiors engaged in safety-specific reward omission and punishment omission behaviors, and answered demographic questions, i.e., gender, age, education, income and seniority. In the second wave survey (T2), which was conducted 2 weeks later, the participants reported their own perceptions of safety-specific distributive justice, safety-specific role ambiguity, and actual safety compliance behaviors. Because of the social distancing of COVID-19 epidemic and the advantage of online survey in collecting sensitive information (53), our questionnaires were distributed to participants on an online survey platform (www.wjx.cn). With the help from the Emergency Management Bureau, we contacted managers of safety management department in the target enterprises, and described the purpose of our survey. After getting their support, research assistants distributed questionnaire to these managers with a quick response code or by hyperlink through WeChat, one of the most popular instant messaging and social interaction application in China and the rest of the world (54), asked them to randomly distribute questionnaires to their front-line workers during weekend time, which ensures that the production activities of front-line workers were not interfered. Participants could fill in the questionnaire from computers as well as from mobile devices. Before filling in the online questionnaire, we informed them that the data would be used for research purposes only and that their personal information would be kept confidential, and asked them to read and understand the purpose of the survey as well as the instructions. Following previous studies like that of Wu et al. (53) we set up one questionnaire for each IP address to prevent more than one completion of each questionnaire.

The data collection lasted for 4 weeks, which is from 20 April to 20 May 2020. In the first wave, the online survey platform distributed 500 questionnaires to front-line workers who are working in high-risk industries. 426 participants filled out and returned, including 32 invalid questionnaires because of missing items, and 394 were retained. In the second wave, 335 out of 394 returned questionnaires, including 28 invalid questionnaires because of missing items and too long or short reaction time, and finally 307 usable questionnaires responses were retained for hypothesis test, yielding a response rate of 61.4%. Table 1 reports the sociodemographic information of the 307 responses.

**Table 1:** Demographic statistics of the sample (*n* = 307).

**Variables**	**Freq**.	**%**	**Variables**	**Freq**.	**%**
**Sectors**			**Gender**		
Construction	111	36.1	Male	250	81.4
Metal-melting	107	34.9	Female	57	18.6
Hazardous chemicals	63	20.5	**Education**		
Machinery manufacturing	26	8.5	Junior high school or below	5	1.6
**Age (year)**			High school or technical secondary school	47	15.3
20–30	95	30.9	Bachelor or senior college	226	73.6
31–40	76	24.8	Master or above	29	9.4
41–50	119	38.8	**Seniority (year)**		
≥51	17	5.5	<1	16	5.2
**Monthly income (RMB)**			[1,2)	24	7.8
≤ 3,000	15	4.9	[2,4)	21	6.8
3,001–5,000	111	36.2	[4,6)	35	11.4
5,001–7,000	116	37.8	[6,10)	36	11.7
7,001–10,000	47	15.3	[10,15)	28	9.1
≥10,000	18	5.9	>15	147	47.9

### Measures

In accordance with the recommendations of previous studies as regards measure contextualization (24, 33, 34), we employed frequently used multi-item scales within a safety context to measure safety-specific leader reward and punishment omission, safety-specific distributive justice, safety-specific role ambiguity, and safety compliance. Since all the scales are English version, we followed appropriate translation and back-translation procedures to ensure the reliability and validity. Specifically, two bilingual experts translated the original scales from English to Chinese in parallel, and two other bilingual scholars conducted a back translation. We then evaluated the semantic equivalence of each back translation, and made adjustments accordingly.

#### Safety-Specific Leader Reward and Punishment Omission

A twelve-item scale for leader reward and punishment omission was adapted from Hinkin and Schriesheim (25). Participants rated the frequency with which their superiors engaged in reward and punishment omission behaviors on a 7-point Likert-type scale (1 = “never” and 7 = “always”). Specifically, six items were developed to assess SLRO, where one sample item is “I often do my jobs safely and still receive no praise from my superior.” The remaining six items were developed to measure SLPO, where one sample item is “When I perform unsafely my superior does nothing.” In the analyses, we excluded one item (i.e., “My unsafety performance often goes unacknowledged by my superior.”) of SLPO due to its low intercorrelations with the other items in the scale.

#### Safety-Specific Distributive Justice

We measured safety-specific distributive justice with a four-item scale derived from Colquitt (55) where the items were adapted to make specific reference to the context of workplace safety. One example item is “To what extent does your reward reflect the effort you have put into your workplace safety?” Participants were asked to report their perception of safety-specific distributive justice on a 7-point Likert-type scale (1 = “to a small extent” and 7 = “to a large extent”).

#### Safety-Specific Role Ambiguity

A five-item scale was derived and adapted from a role ambiguity scale (56) to assess safety-specific role ambiguity by making specific reference to the context of workplace safety. A sample item is “I do not know what my responsibilities are in working safely.” Participants rated their perception of safety role ambiguity on a 7-point Likert-type scale (1 = “strongly disagree” and 7 = “strongly agree”).

#### Followers' Safety Compliance

We assessed followers' safety compliance as the criterion by using a three-item scale adopted from Neal and Griffin (57). One example item is “I use all the necessary safety equipment to do my job.” All items were evaluated on a 7-point Likert-type scale (1 = “strongly disagree” and 7 = “strongly agree).

Control variables. In order to control for alternative explanations of our results, we followed previous research (58–61), and chose employee gender, age, education, income, and seniority as the control variables in this study.

Appendix B summaries all the questionnaires items used in this study.

## Results

### Preliminary Analyses

The common method bias, reliability, and validity were tested before hypotheses testing. First, we used the Harman's single-factor test and confirmatory factor analysis to determine if the variance yielded a single latent factor. Harman's single-factor test indicated that the single factor accounted for 44.73% of the total variance for all measures, far less than the threshold of 70% (62). We further performed confirmatory factor analysis (CFA) to confirm if a single factor accounted for all variables. The results from Mplus 7.4 suggested that the fit of the five-factor model (χ2/*df* = 2.097, CFI = 0.965, TLI = 0.959, SRMR = 0.038, RMSEA = 0.060) was considerably better (Δχ2 = 3360.88, Δdf = 10, p < 0.001) than the single-factor model (χ2/ *df* = 16.617, CFI = 0.473, TLI = 0.420, SRMR = 0.160, RMSEA = 0.226). Taken together, it is reasonable to conclude that common method bias was not a serious concern in this study.

Second, as shown in Table 2, CFA of the scales indicated that the standardized loadings of each construct were higher than 0.70, the composite reliabilities of each construct were near to or larger than 0.90, and the average variance extracted (AVE) exceeded 0.70, showing good convergent validity (63, 64). We also evaluated the discriminant validity by testing whether the square roots of the AVE exceed the corresponding correlations between constructs (64). The results displayed in Table 3 confirmed satisfactory discriminant validity.

**Table 2:** Results of reliability analysis and CFA (*n* = 307).

**Items**	**CFA Loadings**	**Cronbach's α**	**AVE**	**CR**
SLRO1	0.779	0.948	0.755	0.949
SLRO2	0.893			
SLRO3	0.922			
SLRO4	0.882			
SLRO5	0.868			
SLRO6	0.863			
SLPO1	0.752	0.936	0.770	0.943
SLPO2	0.888			
SLPO3	0.810			
SLPO4	0.967			
SLPO5	0.952			
SDJ1	0.792	0.925	0.761	0.927
SDJ2	0.899			
SDJ3	0.903			
SDJ4	0.890			
SRA1	0.922	0.931	0.746	0.936
SRA2	0.897			
SRA3	0.913			
SRA4	0.736			
SRA5	0.838			
SC1	0.815	0.896	0.749	0.899
SC2	0.883			
SC3	0.896			

*SLRO, safety-specific leader reward omission; SLPO, safety-specific leader punishment omission; SDJ, safety-specific distributive justice; SRA, safety-specific role ambiguity; SC, safety compliance; AVE, average variance extracted; CR, composite reliability*.

**Table 3:** Mean, SD, and correlation matrix and square root of AVEs of the study variables.

**Variables**	**Mean**	**SD**	**SEC**	**GED**	**AGE**	**EDU**	**INC**	**SEN**	**SDJ**	**SC**	**SLPO**	**SLRO**	**SRA**
SEC													
GED			−0.13^*^										
AGE			0.42^***^	−0.01									
EDU			−0.33^***^	0.14^*^	−0.36^***^								
INC			−0.30^***^	−0.08	−0.12^*^	0.28^***^							
SEN			0.49^***^	−0.04	0.83^***^	−0.35^***^	−0.04						
SDJ	4.61	1.74	−0.06	0.06	−0.02	−0.04	0.13^*^	−0.06	0.87				
SC	5.73	1.36	0.00	−0.05	0.02	−0.11	0.12^*^	0.04	0.54^***^	0.87			
SLPO	2.47	1.61	0.01	−0.03	0.01	0.14^*^	−0.05	−0.01	−0.32^***^	−0.34^***^	0.88		
SLRO	3.09	1.62	−0.04	−0.03	0.04	0.12^*^	−0.07	0.03	−0.41^***^	−0.37^***^	0.64^***^	0.87	
SRA	1.94	1.09	−0.04	0.01	−0.10	0.10	−0.07	−0.13^*^	−0.33^***^	−0.46^***^	0.40^***^	0.40^***^	0.86

*SEC, sector; GED, gender; AGE, age; EDU, educational level; INC, monthly income; SEN, seniority; SLRO, safety-specific leader reward omission; SLPO, safety-specific leader punishment omission; SDJ, safety-specific distributive justice; SRA, safety role ambiguity; SC, safety compliance; SD, standard deviation. The square roots of AVE are reported in diagonal*;

*** *p < 0.001*;

* *p < 0.05*.

Third, to assess the internal consistencies we used reliability analyses to determine scale Cronbach's alpha. As shown in Table 2, the respective Cronbach's α coefficients for the five scales ranged from 0.896 to 0.948, far greater than the threshold of 0.70 (63), exhibiting a satisfactory reliability of each scales.

Further, Table 3 reports the means, standard deviation, and correlations of all study variables. Overall, respondents reported positive perceptions of safety-specific distributive justice (M = 4.61) and safety-specific compliance behavior (M = 5.73). Also, they showed positive (low) evaluations of safety-specific role ambiguity, SLRO, and SLPO (averaged scores ranged from 1.94 to 3.09). Analyses also showed that the five study variables correlated significantly in expected directions with each other.

### Hypothesis Testing

**Figure 2:**
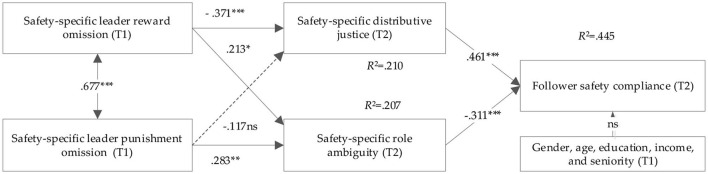
Hypothesized model results. ^***^p < 0.001, ^**^p < 0.01, ^*^p < 0.05, Not significant, *p* > 0.10. T1 and T2 represent the first and second wave data collection, respectively.

We utilized Mplus 7.4 to test the hypotheses (the bias-corrected bootstrapping methodology, with 2,000 resamples at 95% confidence interval) and the results are presented in Figure 2. The hypothesized structural model fits the data well (χ^2^/ df = 1.687, CFI = 0.958, TLI = 0.954, SRMR = 0.055, RMSEA = 0.047). Perceived SLRO shows a negative association with perceived safety-specific distributive justice (*r* = −0.371, *p* < 0.001) and positive association with perceived safety-specific role ambiguity (*r* = 0.213, *p* < 0.001), while SLPO are positively related to followers' perceived safety-specific role ambiguity (*r* = 0.283, *p* < 0.01), supporting H1, H3, and H4. However, the effect of perceived SLPO on perceived safety-specific distributive justice is not significant (*p* > 0.10), hence, not yielding support for H2. As expected, perceived safety-specific distributive justice (*r* = 0.461, *p* < 0.001) and safety-specific role ambiguity (*r* = −0.311, *p* < 0.001) are significantly related to followers' safety compliance, thus supporting H5 and H6.

Further, to test whether perceived safety-specific distributive justice and safety-specific role ambiguity mediated the relationships between SLRO/SLPO and followers' safety compliance, following Preacher and Hayes (65), we used the bias-corrected bootstrapping methodology, with 2,000 resamples at 95% confidence interval, to test the mediation effect with Mplus 7.4 statistic software, because the bias-corrected bootstrapping method can calculate more accurate confidence interval of coefficient product and has higher test power than traditional Sobel method (65, 66). When testing for these mediations, we included the direct relationships between safety-specific leader reward omission and punishment omission, and followers' safety compliance in the model. Table 4 reports the results of the four mediating effects. As we predicted, the indirect effect of perceived safety-specific distributive justice on the relationship between SLRO and followers' perceived safety compliance was significant [−0.147; (−0.236, −0.089), excluding zero], thus supporting H7a. The indirect effects of perceived safety-specific role ambiguity on the relationships between SLRO/SLPO and perceived follower safety compliance were significant, because the 95% confidence intervals were [−0.059; (−0.125, −0.020); and 0.078; (−0.181, −0.050)], excluding zero, thus supporting H8a and H8b. However, the mediating effect of perceived safety-specific distributive justice on the relationship between SLPO and followers' perceived safety compliance (H7b) was not significant [−0.047; (−0.115, 0.006), including zero].

**Table 4:** Mediating effects results.

	**Estimate**	**S.E**.	***p*** **-value**	**Lower 5%**	**Upper 5%**
Mediation of SDJ: SLRO—> SDJ—>SC	−0.147	0.043	0.001	−0.236	−0.089
Mediation of SDJ: SLPO—> SDJ—>SC	−0.047	0.037	0.203	−0.115	0.006
Mediation of SRA: SLRO—> SRA—> SC	−0.059	0.029	0.048	−0.125	−0.020
Mediation of SRA: SLPO—> SRA—> SC	−0.078	0.035	0.026	−0.181	−0.050

*The bias-corrected bootstrapping methodology, with 2,000 resamples at 95% confidence interval, was used to test the mediation effect with Mplus 7.4*.

## Discussion

### Findings

In this study, we partly replicated earlier studies on generalized leader reward and punishment omissions (25, 67) and extended our study by introducing new concepts and measures for safety-specific leader reward omission (SLRO) and a corresponding punishment omission (SLPO) within a laissez-faire leadership framework, as well as adapted context-specific measures of distributive justice and role ambiguity. Further, we retested two mechanisms to explain the relationship between these two domain-specific leadership styles and followers' safety compliance, by employing safety-specific distributive justice and safety-specific role ambiguity as mediators. The results substantiate that SLRO and SLPO are positively associated with followers' safety-specific role ambiguity, which in its turn negatively predicts their safety compliance. Further, SLRO, but not SLPO, is associated with followers' perception of safety-specific distributive justice, which in its turn predicts their safety compliance in a positive direction.

Hence, as regards our two mediation hypotheses, our findings substantiate that followers' safety-specific role ambiguity significantly mediates the relationships between SLRO and SLPO and followers' safety compliance, and that followers' perceived safety-specific distributive justice significantly mediates the relationship between SLRO and followers' safety compliance behaviors. These findings broadly support previous studies in this area linking laissez-faire leadership to followers' safety performance through underlying safety-related cognitive responses (i.e., safety-specific distributive justice and role ambiguity in this study) (6, 8, 9, 16, 21). They also accord with Podsakoff et al. (26) findings showing that followers' perceived distributive justice and role ambiguity mediated the relationship between active-approaching transactional leadership and followers' in-role and extra-role behaviors. However, counter to our hypothesis, and somewhat surprising, the relationship between SLPO and followers' safety compliance is not mediated by their perceived safety-specific distributive justice. However, with second thoughts the present finding can fruitfully be explained by attribution theory (68) which substantiates that followers are likely to attribute their own poor safety performance to external environmental factors beyond their own control (25). Ralph (69) further argued that punishment omission may be perceived more positively by followers because they do not want to be punished when things go wrong. Hence, leaders' non-responses to poor safety performance (i.e., safety-specific leader punishment omission) will probably not be perceived as unfair by followers which, accordingly, explains the insignificant relationship between SLPO and perceived safety-specific distributive justice.

### Implications for Research

This study has important implications for occupational safety research as well as for laissez-faire leadership theory development, the last being an underdeveloped domain in leadership research. First, considering that today's employees, probably, relatively frequently experience detrimental laissez-faire leadership behaviors as compared to the dominating constructive ones (20), and the fact that more superiors are not proactively involved in safety promotion (21), we need more advanced and nuanced empirical studies focusing on plausible mediators in the context-specific laissez-faire leadership—follower safety behavior relationship (15, 17, 21). In this regard, alternative justice forms as well as alternative role stressors are also promising candidates to follow up.

Second, and paradoxically, although previous research shown in Appendix A has extensively examined the roles of safety-specific transformational and active transactional leadership in predicting safety compliance and occupational injuries (8–10, 13–15), to the best of our knowledge, only a few attempts have been made to investigate the potential impact of the contrasting passive-avoidant forms, namely that of context-specific laissez-faire leadership on followers' safety outcomes (15, 17, 21, 32). Hence, this is a strong call to investigate the effects of domain-specific active-approaching forms (cf., safety-specific transactional and transformational forms) in parallel, and in tandem, with domain-specific passive-avoidant forms (cf. safety-specific reward and punishment omissions in this study) to test their unique and combined effects on all those relevant safety outcomes.

Third, beyond prior research examining safety climate as the mediator linking laissez-faire leadership and safety performance, this study introduced justice theory (55) and role theory (30) to the context of occupational safety to offer theoretically grounded explanations on why safety-specific leader reward and punishment omissions are related to followers' safety compliance behaviors through safety-specific distributive justice and safety-specific role ambiguity. Accordingly, and in line with justice theory and role theory, alternative forms of justice (interpersonal, informational and procedural) and role stressors (role conflict and role overload) may fruitfully be tested as mediators in the specified relationships; and in relationships with alternative mediators such as safety climate and with alternative safety outcomes such as safety accidents and injuries.

Fourth, the current study investigated two cognitive mechanisms, but did not consider potential affective mechanisms. In line with the affective event theory by Weiss and Cropanzano (70), followers' positive as well as negative affective responses evoked by leadership behaviors will influence individual compliance decision-making (70–72). Hence, future studies will profit from simultaneously examine both the cognitive and affective mechanisms through which safety-specific leader passive-avoidant and active-approaching leadership behaviors influence followers' safety behaviors.

### Implications for Practices

The findings of the current study have more implications for managerial safety practices. The strongest implication is that leaders' reward and punishment omissions probably decrease followers' safety compliance behaviors, as well as other in-role and extra-role behaviors, through increased followers' perceived safety-specific role ambiguity and reduced safety-oriented distributive justice. This suggests that organizations will profit from inspiring and directing superiors to motivate and stimulate followers' safety compliance with safety-specific contingent reward and punishment, in tandem with safety-specific transformational forms; rather than non-responding to and/or avoiding followers' good or poor safety-related behaviors. Therefore, we believe that it is highly important both to stimulate and reward such leader behaviors for motivating leaders to provide rewards, recognition, and positive feedback contingent on followers efforts to maintain and improve their safety-related behaviors (26, 29). In contrast, punishment or negative feedback should be linked to low or declining levels of safety-related follower performance (29). These differentiated leadership styles, contingent on followers safety behaviors, will probably enable those employees to understand and enact those behaviors which are expected in their daily safety practice, and improve their behaviors thereafter (25, 26, 67). Likewise important, leaders are recommended to administer safety-specific rewards or punishments focusing on the specific safety behaviors that are desirable and undesirable; and not on the individual follower who exhibited those behaviors (29). Furthermore, superiors are suggested to provide timely and personalized rewards and punishments, and match the magnitude of the rewards and punishments to the specific follower behaviors (29); and, in this regard, communicate to followers which safety-specific behaviors will be rewarded and socially approved and which will be non-rewarded and punished (26).

Given the documented direct effects of followers' perceived safety-specific distributive justice and safety-specific role ambiguity on their safety compliance, leaders should remember well and pay even more attention to promote distributive justice and followers' role clarity. For example, in addition to transparent, personalized, and consistent rewards and punishments regimes, systematic and continued feedback seeking may help superiors to collect nuanced information about followers' needs, perceptions, and experienced performances, which can lead to followers' positive cognitive, emotional and behavioral responses via the avoidance of followers' perceptions of unfair safety-specific rewards and punishments (73). Our findings position safety-specific leader reward and punishment omission as a probable ambiguity-increasing leadership style (18, 43, 74), where the withholding and/or avoidance of legitimate and expected leader behaviors, followed by followers ambiguity, have been described to have detrimental consequences (19); further substantiating the necessity to restrain from such leader behaviors.

Furthermore, safety-specific role ambiguity, which is a very stressful demand, will decrease their motivation (75) and, further, trigger anxiety and confusion (76). Thus, we also propose that top managers should focus on reducing followers' safety-specific role ambiguity by elaborating sound policy statements, stimulating “good' safety climate, and clarifying and communicating their expectations to superiors and followers regarding safety performance goals and safe means by which to carry out their tasks safely (18, 26, 74), thus increasing desirable behaviors and outcomes (40).

### Limitations and Future Directions

Although the present study has many strong characteristics, not the least the matched safety-specific measures of all study variables and significant findings, this study has some limitations. First, we used cross-sectional employees' self-reported data to test the hypothesized model, which may lead to common method bias and, further, implies the inability to draw causal conclusions (77, 78). Although the research design and results indicate that the potential common method bias is not a serious threat in the present study, future studies can benefit from collecting evaluations from superiors as well as subordinates, and even the evaluations of superiors' leaders (77, 78).

Another important path to follow in future research is that of reexamining the causal connections examined in the present study by incorporating a timely temporal design (e.g., experimental, longitudinal, and prospective designs) (78). Another limitation concerns the representativeness of the present samples. The present survey study was completed by participants from enterprises from four typical high-risk industries in central China with a satisfactory response rate of 61.4%, higher than the average response rate of 52.7% in organizational research at the individual level (79). Therefore, potential selection biases might have influenced the generalization of our findings. Our recruitment method, however, focusing on industries and employees facing potentially relative high-frequent safety hazards and injuries in their daily work, probably strengthen the generalization of the present study to other comparable industries in China, and even other countries and cultures. As such, more studies are required to replicate the present findings across countries and cultures (80), also taking cultural difference variables into account, which may bolster the relevance and impact of such findings to a broader audience.

## Conclusions

A growing body of studies on occupational safety behaviors has established positive relationships between constructive active-approaching forms of leadership (e.g., safety-specific transformational and active forms of transactional leadership) and followers' safety performance. However, very few studies have examined the degree to which, and how, passive-avoidant forms of leadership influence followers' levels of safety compliance, and no study is found to be focused on safety-specific leader reward and punishment omission (a specific passive-avoidant leadership) and its effect on follower safety compliance. The present study is the first study to investigate the effects of safety-specific leader reward and punishment omission on followers' reported safety compliance, also testing the mediations of followers' safety-specific distributive justice and role ambiguity in a sample of 307 workers from high-risk enterprises in China. We substantiate that safety-specific leader reward and punishment omission will decrease followers' safety compliance behaviors by the attenuation of safety-specific distributive justice and the enhancement of safety-specific role ambiguity, where safety-specific role ambiguity was the stronger mediator by mediating both relationships. Our results suggest that organizations and managers will benefit from recognizing and embracing that supervisors' omissions and avoidances of good and poor safety performance probably have strong negative effects on followers' cognitive responses (e.g., distributive justice and role ambiguity) and their following compliance behaviors. Hence, organizations should take adequate actions, both by firmly disapproving the “dark side” of leaders' safety-related omissions, and by firmly approving and continuously supporting the “bright side” of leaders' safety-related commissions.

## Data Availability Statement

The raw data supporting the conclusions of this article will be made available by the authors, without undue reservation.

## Ethics Statement

The study was approved by the Research Ethics Committee of School of Business at Anhui University of Technology (SB-AHUT-REC-2020-04-HS02 and 03.04.2020). The patients/participants provided their written informed consent to participate in this study.

## Author Contributions

LL contributed to the conceptualization, formal analysis, funding acquisition, investigation, and wrote the first draft. QM contributed to the conceptualization, funding acquisition, and investigation. AS contributed to the conceptualization, formal analysis, and writing—editing. JW contributed to the conceptualization, investigation, and writing—editing. SL contributed to interpretation of the findings and revision of the manuscript. MW made critical revisions of the manuscript. All authors confirmed the final version of the manuscript.

## Funding

This research was supported by the Humanities and Social Sciences Research Projects of the Chinese Ministry of Education [Grant Number 18YJCZH102], the Natural Science Foundation of Anhui Province [Grant Number 2108085QG298], the National Natural Science Foundation of China [Grant Numbers 71874072, 72074099, and 72004081], and the Philosophical and Social Science Foundation of Anhui Province [Grant Number AHSKQ2019D015].

## Conflict of Interest

The authors declare that the research was conducted in the absence of any commercial or financial relationships that could be construed as a potential conflict of interest.

## Publisher's Note

All claims expressed in this article are solely those of the authors and do not necessarily represent those of their affiliated organizations, or those of the publisher, the editors and the reviewers. Any product that may be evaluated in this article, or claim that may be made by its manufacturer, is not guaranteed or endorsed by the publisher.
