# Supplementary material for: Linking Safety-Specific Leader Reward and Punishment Omission to Safety Compliance Behavior: The Role of Distributive Justice and Role Ambiguity
Source: Front Public Health. 2022 Mar 16;10:841345. doi: 10.3389/fpubh.2022.841345 (PMC8966085; doi:10.3389/fpubh.2022.841345)
Supplement: Supplementary file 1 [file Table_1.docx]

**Appendix A: A Summary of Safety leadership studies**

| **Source** | **Leadership type** | **Mediators** | **Outcomes** |
| --- | --- | --- | --- |
| Barling et al., 2002 | Transformational leadership | Safety consciousness, perceived safety climate, and safety-related events | Occupational injuries |
| Zohar, 2002 | Transformational and constructive leadership, and corrective and *laissez-faire leadership* | Safety climate | Behavior-dependent injury |
| Kelloway et al., 2006 | safety-specific transformational and *passive leadership* | Safety consciousness, perceived safety climate, and safety-related events | Occupational injuries |
| Mullen and Kelloway, 2009 | Safety-specific transformational leadership |  | Safety participation, safety compliance, and injuries |
| Clarke, 2013 | Transformational and active transactional leadership | Perceived safety climate | Safety participation, safety compliance, and occupational injuries |
| Hoffmeister et al., 2014 | Transactional leadership (contingent reward and active management-by-exception), and transformational leadership (inspirational motivation, intellectual stimulation, and individualized consideration) |  | Safety compliance, safety participation, and work-related injuries |
| Jiang and Probst, 2016 | Transformational and passive leadership |  | Safety participation |
| Smith et al., 2016 | Safety-specific transformational and *passive leadership* | Safety climate | Safety participation, safety compliance |
| Mullen et al., 2017 | Perceived safety transformational leadership |  | Safety compliance, safety participation |
| Grill et al., 2017 | Transformational, active transactional, rule-oriented, participative, and *laissez-faire leadership* |  | Safety climate, safety behavior, and accidents |
| Xue et al., 2020 | Transformational safety leadership (safety inspiration, safety concern, safety vision, and personal character), transactional safety leadership (safety policy and safety awards and punishment) | Safety climate | Safety participation, safety compliance |

Note: A relatively few studies focus on laissez-faire leadership and no study explicitly focuses on safety-specific leader reward and punishment omission. Among limited studies examining the underlying mechanism, safety climate is considered to be the mediator linking leadership and safety performance.

**Appendix B: Questionnaires items.**

| **Constructs** | **Scale Items** | **Sources** |
| --- | --- | --- |
| Safety-specific Leader Reward Omission | I often do my jobs safely and still receive no praise from my manager. | Adapted from Hinkin &Schriesheim (2008) |
|  | My manager often gives me no feedback when I do my jobs safely. |  |
|  | When I do my jobs safely my manager usually does nothing. |  |
|  | My safety performance often goes unacknowledged by my manager. |  |
|  | I don’t often get praised by my manager when I perform safely. |  |
|  | My safety performance often gets no response from my manager. |  |
| Safety-specific Leader Punishment Omission | I seldom get criticized by my manager when I perform unsafely. | Adapted from Hinkin &Schriesheim (2008) |
|  | My manager gives me no feedback when I perform unsafely. |  |
|  | When I perform unsafely in my job I receive no criticism from my manager. |  |
|  | When I perform unsafely my manager does nothing. |  |
|  | My unsafety performance often gets no response from my manager. |  |
|  | My unsafety performance often goes unacknowledged by my superior. |  |
| Safety-specific Distributive Justice | To what extent does your reward reflect the effort you have put into your workplace safety? | Adapted from Colquitt (2001) |
|  | To what extent is your reward appropriate for the work you have completed safely? |  |
|  | To what extent does your reward reflect what you have contributed to your workplace safety? |  |
|  | To what extent is your reward justified, given your safety performance? |  |
| Safety-specific Role Ambiguity | I do not have clear planned safety goals and objectives for my job. | Adapted from Peterson et al., (1995) |
|  | I do not know exactly what is expected of my safety behaviors. |  |
|  | I do not know what my responsibilities are in working safely. |  |
|  | I do not feel certain about how much safety responsibility I have. |  |
|  | My safety responsibilities are not clearly defined. |  |
| Followers’ Safety Compliance | I used all the necessary safety equipment to do my job in past week. | Neal & Griffin (2006) |
|  | I used the correct safety procedures for carrying out my job in past week. |  |
|  | I ensured the highest levels of safety when I carry out my job in past week. |  |
